# Blastic Plasmacytoid Dendritic Cell Neoplasm: First Case Report From Rwanda and Review of the Literature

**DOI:** 10.1200/JGO.19.00123

**Published:** 2019-06-27

**Authors:** Deogratias Ruhangaza, Marcellin C. Mugabe, Catherine N. Kigonya, Andrew A. Lane, Elizabeth A. Morgan

**Affiliations:** ^1^ Butaro Cancer Centre of Excellence, Butaro, Rwanda; ^2^ Dana-Farber Cancer Institute, Boston, MA; ^3^ Brigham and Women’s Hospital, Boston, MA

## INTRODUCTION

Blastic plasmacytoid dendritic cell neoplasm (BPDCN) is a rare and aggressive hematologic malignancy that typically presents as one or more cutaneous lesions with or without associated bone marrow involvement. Classification has evolved over time with several different names (CD4+ acute agranular natural killer [NK]–cell leukemia, blastic NK-cell lymphoma, blastic NK leukemia/lymphoma, agranular CD4+ CD56+ hematodermic neoplasm/tumor) until it was discovered that this neoplasm is derived from plasmacytoid dendritic cells.^1^ The BPDCN nomenclature was adopted in the 2008 World Health Organization Classification of Tumours of Haematopoietic and Lymphoid Tissues under the category “Acute myeloid leukaemia and related precursor neoplasms,”^2^ and BPDCN is listed as its own category in the 2016 World Health Organization revision.^3^ Herein, we present what is to our knowledge the first reported case of BPDCN in a Rwandan patient.

## CASE REPORT

### Clinical Presentation

**FIG 1:**
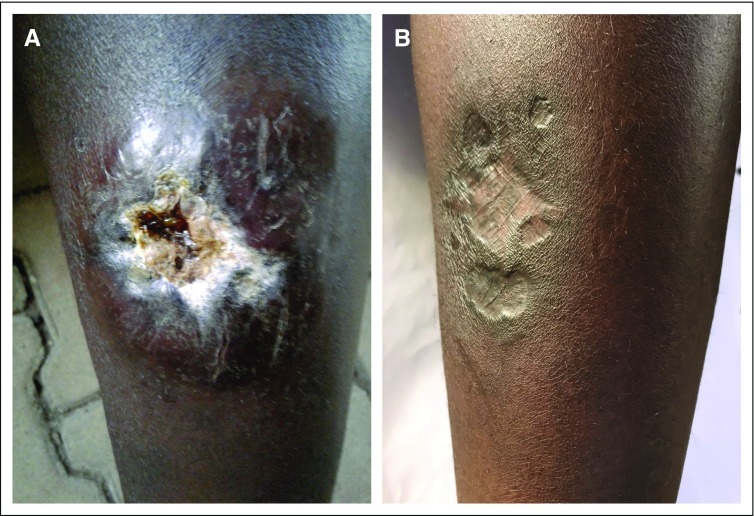
Cutaneous lesion before and after induction therapy. (A) Ulcerated skin tumor before starting treatment, (B) completely healed skin lesion at the interim maintenance phase.

A 29-year-old woman presented with a 2-year history of a slow-growing, indurated, and ulcerated skin mass at the anterior medial aspect of the left lower leg that was tender and mobile. The mass measured 5 × 6 cm, with skin surface ulceration, purulent drainage, and foul smell, probably as the result of wound superinfection (Fig 1A). The patient had no significant medical history. A routine blood count was within normal limits (WBC: 7,500/μL; hemoglobin: 13.8 g/dL; platelets: 311,000/μL). The patient initially consulted traditional healers, without improvement. A biopsy of the lesion was performed at Butaro Cancer Centre of Excellence (Butaro, Rwanda) and sent to Brigham and Women’s Hospital (Boston, MA) for additional work-up.

### Diagnosis

**FIG 2:**
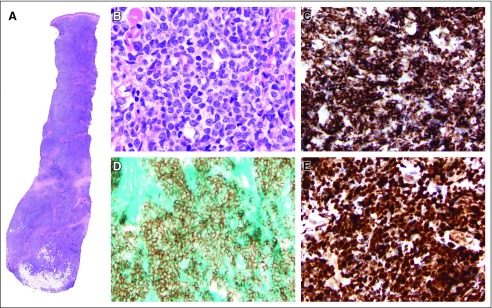
Histologic and immunohistochemical features of the tumor. (A) Low-power view of the skin incisional biopsy demonstrating a dense dermal infiltrate extending into the subcutis. The overlying epidermis is uninvolved. Hematoxylin and eosin; magnification, ×10. (B) Higher-power view showing an infiltrate of intermediate- to large-sized immature cells with round to irregular nuclei, dispersed chromatin, distinct small nucleoli, and scanty cytoplasm. Hematoxylin and eosin; magnification, ×1,000. The tumor cells are diffusely positive for CD56 (C), CD123 (D), and TCL1 (E). Magnification, ×500.

Tissue sections showed a deep skin incisional biopsy, extending to the subcutis (Fig 2A). The dermis and subcutis were diffusely infiltrated by a monotonous population of intermediate- to large-sized immature cells with round to irregular nuclei, dispersed chromatin, distinct small nucleoli, and scanty cytoplasm (Fig 2B). Frequent mitotic figures were observed. The overlying epidermis was not involved. An initial limited panel of immunostains was performed at the Butaro District Hospital Pathology Department, demonstrating that lesional cells were positive for CD45 (diffuse), terminal deoxynucleotidyl transferase (majority), and PAX5 (weak, small subset); lesional cells were negative for CD3, CD20, myeloperoxidase, and lysozyme. Given the inconclusive immunophenotype, the case was sent to Brigham and Women’s Hospital for additional immunostains. These additional studies revealed that lesional cells were positive for CD2, CD33, CD4 (weak), CD56 (Fig 2C), CD123 (Fig 2D), and TCL1 (Fig 2E); lesional cells were negative for CD10, CD19, CD34, CD7, and CD5. On the basis of morphologic and immunohistochemical findings, a diagnosis of BPDCN was rendered. A staging bone marrow biopsy was not performed before treatment initiation. A bone marrow biopsy performed after the induction phase of therapy revealed a hypocellular marrow (30% cellular) with maturing trilineage hematopoiesis and no morphologic evidence of disease. A 95-gene sequencing panel showed no pathogenic single-nucleotide variants or small insertions/deletions, although several variants of unknown significance were reported (ATM c.1810C>T p.P604S [in 58.9% of 440 reads]; CREBBP c.7306G>A p.E2436K [in 8.2% of 220 reads]; NOTCH3 c.4469_4472CGG​A>GCG​C p.1490_1491delTEinsRA [in 11.3% of 133 reads]).^4^

### Treatment and Outcome

Because of her young age and otherwise excellent performance status, our patient started receiving an intensive systemic chemotherapy regimen used to treat acute lymphoblastic leukemia (ALL), with intrathecal therapy for CNS prophylaxis. The treatment regimen we chose (Table 1) is a modified treatment for ALL that was proposed by Hunger et al^5^ specifically for low-resource settings. It consists of initial and delayed aggressive phases of therapy (induction/consolidation and delayed intensification) with an intervening less intense phase of treatment known as interim maintenance. After completion of these blocks of more aggressive therapy, which generally takes between 6 and 9 months, there is a prolonged period of maintenance therapy that lasts 2 years. Our patient is currently in the interim maintenance phase and has responded well, showing complete healing of the skin tumor (Fig 1B).

**CONTEXT**

**Key Objective**
How do we approach the diagnosis and treatment of a patient with BPDCN in a resource-limited setting?
**Knowledge Generated**
The diagnosis and treatment of a young patient in Rwanda with BPDCN required a collaborative effort between the local cancer center in Rwanda and an academic medical center in the United States. Accurate diagnosis and a tailored therapeutic regimen resulted in a significant clinical response.
**Relevance**
This report highlights the key features of a rare hematopoietic malignancy and emphasizes the benefits of global health clinicopathologic consultations.

**TABLE 1:**
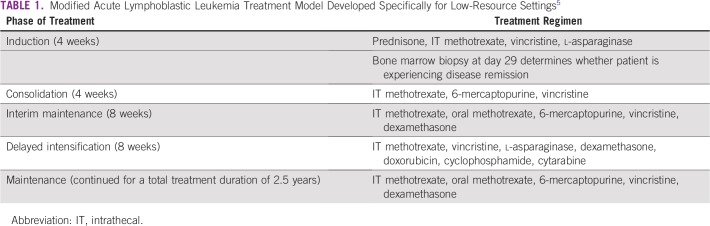
Modified Acute Lymphoblastic Leukemia Treatment Model Developed Specifically for Low-Resource Settings^5^

## DISCUSSION

BPDCN is a rare hematologic malignancy thought to be related to myeloid disease.^6^ BPDCN is most common in older patients with a mean age at diagnosis of 67 years (range, 8 to 103 years) and a significant male predominance.^7^ Our patient was a young female, making clinical suspicion for this disease difficult. BPDCN may occur in the context of other hematologic malignancies (particularly myelodysplastic syndromes, acute myeloid leukemia, and chronic myelomonocytic leukemia) in up to 10% of cases.^8^

BPDCN manifests as cutaneous lesions with or without associated bone marrow involvement. Skin lesions can appear as plaques or tumors that can be isolated or widespread. Some patients may exhibit striking plum-colored or bruise-like patches and plaques that evolve to violaceous, dome-shaped tumors covered by a shiny, attenuated epidermal layer. Splenomegaly and lymphadenopathies are also common.^8^ Leukemic involvement is often associated with pancytopenia; patients rarely present with marrow-only disease lacking cutaneous manifestations.^9^

Skin, lymph node, and bone marrow biopsies demonstrate diffuse infiltrates of monomorphic intermediate- to large-sized blasts with scanty cytoplasm, round or oval folded nuclei, fine chromatin, and two to three small nucleoli.^10^ Positive markers include CD4, CD56, CD123 (interleukin-3 receptor alpha-chain), TCL1, and BDCA-2/CD303 (blood dendritic cell antigen-2).^11^ Terminal deoxynucleotidyl transferase expression is detected in up to 40% of cases, as observed in our patient, whereas other markers of immaturity (eg, CD117, CD34) are nearly always negative.^12^ The absence of lineage-defining markers (eg, CD19, CD3, myeloperoxidase) should be a helpful clue to consider this diagnosis, but the expression in BPDCN of other markers commonly associated with B-cell (CD79a), T-cell (CD7, CD2, CD5), or myeloid (CD33, CD68) origin can be confounding. The presence of PAX5 expression, albeit subset weak, was particularly confusing in the initial work-up of this lesion and, to our knowledge, has not been described in BPDCN.

Several recurrent genetic abnormalities have been discovered in BPDCN, but there is no single cytogenetic change that is specific or diagnostic.^6,13,14^ Karyotypic abnormalities include mainly loss of genetic material, with certain chromosomes targeted (15q, 5q, 13q, 6q, and 12p). Monoallelic deletion of the NR3C1 locus at 5q31 was identified in 28% of patients and is associated with poor outcome.^15^ Recurrent *MYB* gene rearrangements with variable translocation partners have been described in 100% of pediatric cases (n = 5) and 44% of adult cases (n = 4) in one study.^16^ Recurrently mutated genes include *TET2* (most common), *NPM1*, *ASXL1*, *NRAS*, *IDH2*, *APC*, *ATM*, *ZRSR2*, *SRSF2*, *SF3B1*, *U2AF1*, *SF3A2*, *SF3B4*, *TP53*, *GNB1*, *ETV6*, *DNMT3A*, *RUNX1*, *CRIPAK*, *NEFH*, *HNF1A*, *PAX3*, and *SSC5D*.^6,14,17^ In our patient, genetic testing of the skin lesion was not performed. Mutational analysis of the uninvolved bone marrow biopsy specimen, which was obtained after the initiation of therapy, did not show recurrent pathogenic mutations but did show several variants of unknown significance, with variant allele frequencies ranging from 8.2% to 58.9%. We previously demonstrated that in older adult patients, clonal hematopoiesis is detected at a high variant allele frequency in bone marrow samples that lack or have minimal morphologic evidence of involvement by BPDCN, a phenomenon that persists after therapy.^18^ However, the significance of variants detected in our patient remains uncertain.

The differential diagnosis of BPDCN includes other conditions with more mature plasmacytoid dendritic cells such as Kikuchi-Fujimoto disease and chronic myelomonocytic leukemia. In these conditions, mature plasmacytoid dendritic cells lack CD56, and their presence is not indicative of involvement by BPDCN. Other entities within the differential include CD4+/CD56+ diseases that can present in the skin, but extensive immunophenotyping should allow them to be distinguished from BPDCN. Examples include nasal-type extranodal NK/T-cell lymphoma which, unlike BPDCN, will express for Epstein-Barr virus–encoded small RNAs by in situ hybridization, or cutaneous monocytic leukemias, which will express lysozyme.

The optimal treatment of BPDCN is not well established because of insufficient data. In retrospective series, more intensive, acute leukemia–like induction chemotherapy and ALL-type regimens (as compared with AML- or lymphoma-type regimens) are associated with improved outcomes in BPDCN.^19,20^ These studies informed our treatment strategy in this case. Most adult patients for whom complete or partial remission is achieved experience disease relapse within 2 years after induction therapy. Hematopoietic-cell transplantation is recommended for adults in first complete remission,^21^ but this is not feasible in our resource-limited setting.

As disease-specific clinical trials are performed, novel therapies are emerging for BPDCN. In 2018, the US Food and Drug Administration approved tagraxofusp (SL-401), an interleukin 3–diphtheria toxin fusion protein, for treatment of patients with BPDCN.^22,23^ Treatment with the BCL-2 inhibitor venetoclax also showed significant disease response in two patients with relapsed/refractory BPDCN.^24^ Targeted therapies such as these may eventually become attractive alternatives to systemic chemotherapy in resource-limited settings.^25^ In pediatric patients, systematic literature review has highlighted a better initial response to therapy compared with adults, with a better overall survival rate and lower rates of relapse.^26^ Younger age and isolated cutaneous disease are reported to be associated with better prognosis.^8,27^ Our patient remains well, without evidence of disease, 10 months after diagnosis.

In addition to contributing to our knowledge of the clinicopathologic features of BPDCN, this case report is an excellent demonstration of the collaborative relationship between pathologists and clinicians at Butaro District Hospital/Cancer Centre of Excellence in Rwanda and Brigham and Women’s Hospital/Dana-Farber Cancer Institute in Boston.^28,29^ The pre-existing pipelines for telepathology and transfer of materials between the institutions facilitated rapid consultation of this unusual hematopathology specimen, which could not have been diagnosed in Butaro given the requirement for specialized immunohistochemical antibodies. After the diagnosis was rendered, the established and open channels of communication between the institutions allowed clinicians with complementary areas of expertise (BPDCN treatment in Boston; cancer care in resource-limited settings in Butaro) to formulate an appropriate therapeutic plan. This joint effort, which emphasizes cooperation and consultation, enriched the education of all involved physicians and contributed to a successful patient outcome.

We have presented, to our knowledge, the first described case of a rare malignancy, BPDCN, in Rwanda. We have highlighted the challenges in diagnosis and treatment of this condition, especially in our resource-limited setting. We hope that this case report adds to knowledge and awareness of this rare hematologic malignancy, particularly in younger patients.
